# Epidural Analgesia and Neonatal Morbidity: A Retrospective Cohort Study

**DOI:** 10.3390/ijerph15102092

**Published:** 2018-09-24

**Authors:** Antonio Hernández Martínez, Julián Javier Rodríguez Almagro, María Moreno-Cid García-Suelto, María Ureña Barrajon, Milagros Molina Alarcón, Juan Gómez-Salgado

**Affiliations:** 1 Midwife Unit, Mancha-Centro Hospital, 13600 Alcázar de San Juan, Ciudad Real, Spain; antomatron@gmail.com (A.H.M.); unidadmujeralcazar@quironsalud.es (M.M.-C.G.-S.); jupij79@gmail.com (M.U.B.); 2 Department of Emergency, Ciudad Real University Hospital, 13005 Ciudad Real, Ciudad Real, Spain; 3 Department of Nursing, Faculty of Nursing, University of Castilla la Mancha, 02071 Albacete, Spain; Milagros.molina@uclm.es; 4 Department of Nursing, Faculty of Nursing, University of Huelva, 21071 Huelva, Spain; jgsalgad@gmail.com; 5 Espíritu Santo University, Guayaquil 092301, Ecuador

**Keywords:** Apgar, epidural analgesia, neonatal morbidity, neonatal resuscitation, umbilical artery pH

## Abstract

(1) *Background*: Epidural analgesia (EA), at the present time, is one of the most effective methods to reduce labor pain. In recent years its use has increased, being used between 20–70% of all deliveries; (2) *Methods*: Historical cohort on a total of 2947 deliveries during the years 2012–2016 at the “Mancha-Centro Hospital” of Alcázar de San Juan. The main outcome variables were four neonatal morbidity (NM) criteria: umbilical artery pH of <7.10, Apgar score at 5 min < 7, need for advanced resuscitation and composite morbidity. We used the multivariate analysis to control confounding bias. (3) *Results*: No statistical relationship between EA and the second stage of labor duration with none of the four criteria of NM used (*p* > 0.005). However, the type of delivery was associated with three criteria (pH, resuscitation, and composite morbidity). The instrumental delivery presented an OR of pH < 7.10 of 2.68 95% CI [1.15, 6.27], an OR of advanced resuscitation of 2.44 95% CI [1.17, 5.08] and OR of composite morbidity of 2.86 95% CI [1.59, 5.12]; (4) *Conclusions*: The EA and the second stage of labor duration are not related to the NM. While the instrumental delivery doubles the risk of NM compared to the normal vaginal delivery.

## 1. Introduction

Epidural analgesia (EA), at the present time, is one of the most effective methods to reduce labor pain [1]. In recent years its use has increased, being used between 20–70% of all deliveries [2,3,4,5,6].

At the same time, several studies have described various adverse effects among pregnant women users of EA, such as rise in body temperature, difficulty in the onset of lactation, hypotension, prolonged second stage of labor, and an increase in instrumental vaginal delivery, among others [1,6,7,8,9,10,11,12].

In terms of its effect on the newborn, the main systematic reviews have found no relationship between the use of EA and an increase in neonatal morbidity (NM), usually expressed by means of low Apgar scores and pH values of umbilical artery [1,6,10,13,14,15].

However, in recent years several works have been published that relate the use of EA with an increase in NM [3,5,16,17,18]. These works can negatively influence professionals and pregnant women when using EA, as it leads them to believe that EA could be potentially dangerous for the newborns.

Especially, it is not clear whether this increase in morbidity occurs by direct effect of EA or as a result of its potential adverse effects as is the increase of instrumental vaginal delivery [4,19] or prolonged second stage of labor [16,17,20]. In addition, some of these works have major methodological limitations by an inappropriate design, lack of control of confounding bias or unclear or unjustified exclusion criteria. These limitations cast doubt on the true relationship between the use of EA and neonatal morbidity.

Therefore, the objective of our study was to determine the relationship between the use of EA and NM, with the purpose of clarifying whether EA is a risk factor or other factors associated with their use are the real culprits.

## 2. Materials and Methods

The study was conducted in accordance with the principles of the Declaration of Helsinki and approved by the ethical committee of institutional review board of Hospital Mancha Centro code number 69-C in October 2017.

### 2.1. Design and Participants

The nature of study is observational, analytical, retrospective cohort. It has been done in the delivery services of the Mancha-Centro District Hospital in Alcázar de San Juan.

**Figure 1:**
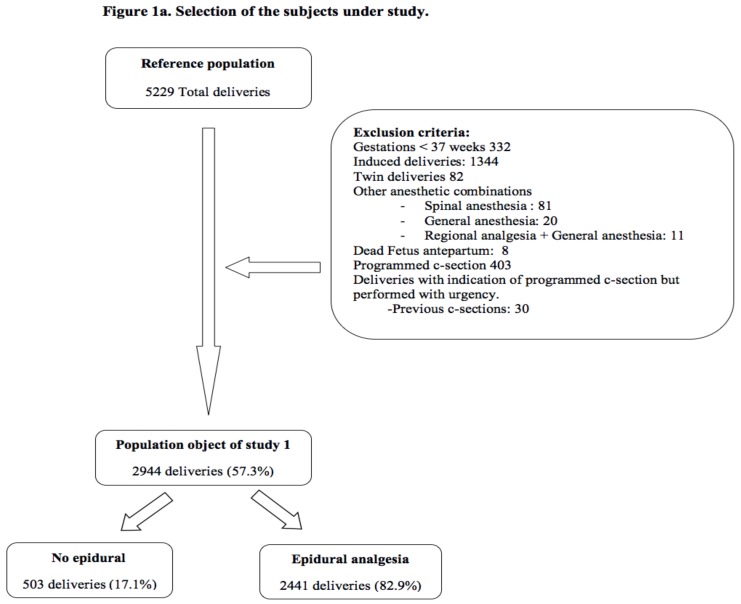

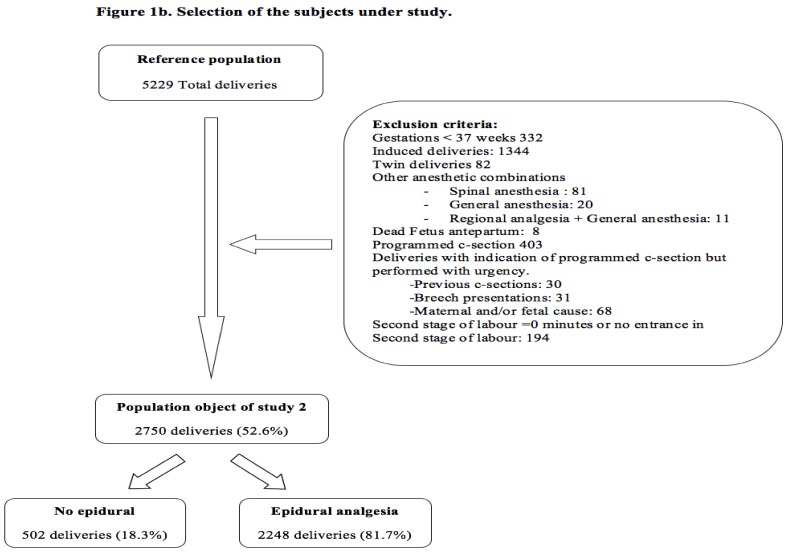
Selection of the subjects under study. (**a**) Population 1. Selection of the subjects under study. (**b**) Population 2. Selection of the subjects under study.

The reference population is composed of a group of pregnant women who receive care during childbirth in the Mancha-Centro District Hospital, from 2012 to 2016. All planned surgical deliveries (caesarean sections), gestations under 37 weeks, induced delivery, twin gestations, and other combinations of analgesic techniques other than the exclusive use of EA were excluded. We excluded other analgesic combinations so as to know the influence of epidural analgesia and to avoid confounding bias. With these criteria of exclusion, the study population 1 was staked out (Figure 1a). But due to the duration of the second stage of labor is a potential risk factor for NM and many pregnant women did not reach the second stage of labor because they underwent a caesarean section in the first stage of labor, a study population 2 was created where pregnant women who did not reach the second stage of labor were excluded (Figure 1b).

The mothers were, in both populations, divided between those who received EA for the relief of pain during delivery and those who did not receive it. The administration of EA in our center was continuous through an infusion pump with a concentration of 1mg/mL of ropivacaine and 2 mcg of fentanyl, managed to pace between 12–14 mL/h depending on needs of the patient.

To estimate the sample size, we consider as the main event the pH of the umbilical artery to be an objective criterion and as a cut off [21,22], values less than 7.10. [23] Considering that the average number of deliveries a year is 1100 and that around 3% may have pH values below 7.10, we would have nearly 33 newborns a year.

To build a multivariate model, ten events are required (pH under 7.10) to add each variable. Assuming a maximum of ten variables in the initial model, we would need three years of data.

### 2.2. Sources of Information

To collect the data, we have used the medical records of the patients under study.

The following variables were collected:

Main outcome variables: pH umbilical artery <7.10, Apgar score after 5 min <7, degree of neonatal resuscitation (basic/advanced) and composite morbidity (created on the basis of the other three variables) [24,25]. Neonatal resuscitation was considered basic when required drying and temperature maintenance, oronasopharyngeal suction and indirect administration of oxygen. The resuscitation was considered advanced when oxygen was administered with positive pressure, intubation, cardiac massage or use of drugs.

Main independent variables: use of epidural analgesia.

The control variables were:

Maternal age, gestational age, body max index (BMI), previous caesarean section, type of pregnancy, parity, type of delivery, first stage of labor duration, second stage of labor duration, and birth weight.

### 2.3. Statistical Analysis

First of all, a descriptive statistics analysis was performed using absolute and relative frequencies for categorical variables and arithmetic means and standard deviation for quantitative ones. 

Then, bivariate analysis was performed on the obstetric history, development, and outcome of labor and neonatal morbidity with the use of EA (No/Yes), using the chi-square test for categorical variables and Student’s *t*-test for quantitative variables.

To determine the risk of NM, multivariate analysis was performed using binary logistic regression. The aim of the model was to determine the clear effect of the use of EA controlling potential confounding factors. A first analysis was carried out on the population under study 1 and then on the object of study population 2. We estimated the odds ratio (OR) with their respective confidence intervals (CI) of 95%, using as a reference category the most physiological or normal option. For the statistical analysis, the software SPSS v20.0 was used. (SPSS Inc., Chicago, IL, USA).

## 3. Results

The reference population consists of a total 5229 pregnant women, of whom 2622 (44.7%) were excluded for various reasons, participating definitely 2944 pregnant women (57.3%) in population 1 (Figure 1a) and 2750 (52.3%) in population 2 (Figure 1b).

The use of EA within population 1 stood at 82.9% (2441) and in population 2 at 81.7% (2248).

As for the main maternal and obstetric characteristics and their relation to the use of EA, a statistically significant association was observed with: gestational age, parity, duration of dilatation, duration of the second stage of labor and type of delivery. No relationship was found between maternal age, BMI, and birth weight. Obstetric characteristics are detailed in Table 1.

We also studied the relationship between EA and NM in population 1, and found no statistically significant differences between values in umbilical artery pH < 7.10 (1.4% vs. 1.8%; *p* = 0.831), Apgar scores <7 at 5 min (0.0% vs. 0.4%; *p* = 0.993), both in the univariate analysis and adjusting for confounding factors. In the case of the degree of neonatal resuscitation, statistical association was observed in the univariate analysis with EA (0.0% vs. 2.9%; *p* < 0.05), but not the adjusted model (*p* = 0.993). The same situation was observed with the composite morbidity variable, where the statistical association reflects univariate analysis (1.2% vs. 4.2%; *p* < 0.001), but not the adjusted model (OR: 1.97 CI 95% [0.82, 4.74]). Data on NM and EA are shown in Table 2.

The next step was to analyse the relationship between use of EA, type of delivery and duration of the second stage of labor in those pregnant women who reach the second stage of labor. In Table 3 it can be seen how only 0.4% (2) of the pregnant women who do not use EA have a second stage of labor greater than 3 h, while between the users of EA overcame this period at 8.8% (177) of the normal vaginal deliveries, 34.5% (67) of the instrumentals and 44.2% (22) of caesarean sections (*p* < 0.001).

Finally, we studied the relation between EA, type of delivery, and duration of the second stage of labor with NM in population 2, finding no statistically significant differences between EA and duration of the second stage of labor and the four criteria of NM used. On the other hand, the type of delivery was statistically associated with three criteria (pH, neonatal resuscitation and composite morbidity). Specifically, the instrumental delivery presented an OR of pH < 7.10 of 2.68 95% CI [1.15, 6.27], an OR of advanced neonatal resuscitation of 2.44 95% CI [1.17, 5.08] and OR composite morbidity of 2.86 95% CI [1.59, 5.12]. The delivery by caesarean section in this population was not related to any of the criteria of NM used (Table 4).

## 4. Discussion

In this study, to know if the use of EA is associated with an increased NM, no relation between the two variables or with the duration of the second stage of labor was found. However, the instrumental delivery was associated with an increased risk of NM.

The prevalence of use of EA in this work was above 80%, a figure that we can consider high if compared with the most recent studies about its influence on NM. [2,3,4,16,19,26]. Only the study of Laughon [17] found similar rates of use. The group of women who receive epidural is fivefold bigger than those who do not receive it. However, this is the existing difference in real practice in our center and in many Spanish centers [27].

These differences in use can affect the subsequent results, as the observational work where the use of EA is low, concentrate pregnant women with an obstetric profile of greater risk in this group (nulliparous, [3,4,19,28,29] fetuses of greater size, [29] etc.). For all these reasons, it is necessary that the observational studies establish confounding control techniques appropriate on the main risk factors for NM. 

In this sense, the relationship between EA and NM using multivariate analysis was studied. The NM was assessed through four criteria: Apgar scores <7 at 5 min, artery pH < 7.10, higher needs of neonatal resuscitation and composite morbidity. We have used this composite outcome as we believe this is a global summarizing result that includes objective criteria (umbilical artery pH) and subjective criteria (Apgar and degree of resuscitation), in addition to its previous use by other authors. [24,25].

Also, the relationship between the EA and NM in two different populations was studied: the population 1 (which includes all the pregnant women regardless of the fact that they could reach the second stage of labor) and the population 2, (where those that did not reach the second stage of labor were excluded) without observing association between EA and NM. These results coincide in a comprehensive manner with various revisions [6,10,14] and original works, [4,7,8,19,20,26,28,29,30] although there is an important variability in the criteria and cut off points used for the valuation of NM.

On the contrary, some authors have observed an increase in low Apgar scores, [3,5,16,17,18] lower values of pH in normal vaginal deliveries [31] and higher needs of neonatal resuscitation among users of EA. [3]. Even so, their results in terms of mortality and hypoxic-ischemic encephalopathy in the neonates to study are not unanimous [5,17].

Moreover, these publications present important limitations, in regard to the information on whether there is confounding control in the analysis of the influence of EA [3,18,31] using in some cases averages of scores instead of cut-off points, finding statistical differences but little clinically relevant [3,32].

Another remarkable result is that the duration of the second stage of labor, although it is closely related to the use of EA and at the same time with the type of delivery, it was not associated with greater NM either. This result is consistent with the work of Cheng, [2] while other authors have found an increasing risk of NM in prolonged second labor stages. [16,17]. These discrepancies are justified by the variability in the control systems of the fetal well-being during delivery and the protocols of work of each center. All this justifies the need for further studies on the influence of the duration of the second stage of labor in NM, in order to establish recommendations as to the limits of its duration.

However, the type of delivery, specifically the instrumental delivery, is related to greater NM. The instrumental deliveries doubled the morbidity of the normal vaginal deliveries in three of the four criteria used. Hasegawa [19] and Hung [4] concurred with our findings, concluding that the instrumental delivery could itself be more responsible for the NM than EA. Curiously, the deliveries that ended in caesarean did not present higher NM than the normal vaginal delivery. These results may be due to the fact that the pregnant women that undergo a caesarean section, in spite of presenting a percentage of more prolonged deliveries with expulsive, are carried out under conditions of fetal well-being better than when an instrumental vaginal delivery is indicated. The impact of instrumental delivery is more possibly a reflection of a (suspected) clinical fetal or maternal condition, not so much a cause of poor neonatal outcome. The instrumental vaginal delivery in the second stage of labor is a faster option for the removal of a compromised fetus, so it is possible that this is not a risk factor for the NM in many deliveries, but the maternal-fetal situation that determines the clinician to the practice of this. In addition, women who receive epidural analgesia present a higher risk of having an instrumental delivery due to the specific characteristics of women who demand epidural use (nulliparous, [3,4,19,28,29] fetuses of greater size, [29], etc.).

Due to the nature of the study, it has its limitations, which are inherent to retrospective studies and may be responsible for certain biases. As a limitation of our work, we find a significant amount of lost pH values. However, we believe that these values were not collected as they were related to deliveries without complications that allowed for the delayed clamping of the umbilical cord. As the cases collapsed, the extraction of the sample was not possible. Another aspect that could be improved was the lack of knowledge, due to the retrospective nature of the work, whether the use of EA was associated with a more effective maternal breastfeeding in the early hours.

As a strength, we can highlight the analytical techniques for confounding control used that allow us to isolate the clear effect of the use of EA on neonatal morbidity, the use of the pH value as an objective criterion, the use of the composite morbidity variable that integrates various results of morbidity and the large sample size that was used.

## 5. Conclusions

Finally, we can conclude that our study did not see a relationship between the use of EA and the indicators of NM. However, it is necessary to develop new studies to know the influence of EA in the longer term, to set the temporary limits of a safe second stage of labor, and the development of new strategies to reduce the practice of instrumental deliveries or can be carried out in better clinical conditions.

## Figures and Tables

**Table 1:** Associated factors to epidural analgesia that can be presented as potential confounding factors in population 1.

Variables	Epidural Analgesia		*p*-Value
	No (*n* = 503)	Yes (*n* = 2441)	
Maternal Age			0.665
Mean (SD)	30.8 (5.67)	30.7 (5.20)	
Gestational Age			<0.001
Mean (SD)	39.5 (1.07)	39.8 (1.06)	
Parity			<0.001
Nulliparous	112 (22.3)	1348 (55.2)	
Multiparous	391 (77.7)	1093 (44.8)	
Previous CS			0.137
No	478 (95.0)	2276 (93.2)	
Yes	25 (5.0)	165 (6.8)	
BMI			
Mean (SD)	24.7 (4.60)	24.4 (4.54)	0.579
Newborn weight (grams)			0.051
Mean (SD)	3271.8 (444.82)	3313.8 (412.01)	
First stage of labor duration (minutes)			<0.001
Mean (SD)	94.4 (97.19)	270.5 (161.56)	
Second stage of labor duration (minutes)			<0.001
Mean (SD)	19.3 (23.12)	78.4 (63.37)	
Type of delivery			<0.001
Normal Vaginal	503 (100.0)	2006 (82.2)	
Instrumental	0 (0.0)	194 (7.9)	
Emergency CS	0 (0.0)	241 (9.9)	

BMI = Body Mass Index; CS = Caesarean Section; EA = Analgesia Epidural; SD = Standard Deviation.

**Table 2:** Main key indicators of neonatal morbidity and its relation with the use of epidural analgesia in population 1.

Variables	Epidural Analgesia				*p*- Value Aj *
	No (*n* = 503) *n* (%)	Yes (*n* = 2441) *n* (%)	OR Crude	OR Adjusted *	
Umbilical artery pH					0.831
≥7.10 (Ref)	425 (98.6)	2164 (98.2)	1.00	1.00	
<7.10	6 (1.4)	39 (1.8)	1.27 [0.54, 2.99]	0.78 [0.31, 1.96]	
Missing	72	238			
Apgar at 5 min					0.993
≥7 (Ref)	503 (100.0)	2430 (99.6)	1.00	1.00	
<7	0 (0.0)	10 (0.4)	NC	NC	
Missing	0	0			
Degree of neonatal resuscitation					0.992
Basic (Ref)	503 (100.0)	2330 (97.1)	1.00	1.00	
Advanced	0 (0.0)	71 (2.9)	NC	NC	
Missing	0	0			
Composite morbidity					0.132
No (Ref)	497 (98.8)	2338 (95.8)	1.00	1.00	
Yes	6 (1.2)	103 (4.2)	3.65 [1.59, 8.36]	1.97 [0.82, 4.74]	
Missing	0	0			

Ref = Reference category. * Aj. Odds Ratio adjusted with binary logistic regression by maternal age, birthweight, gestational age, parity, type of delivery, previous caesarean section, first stage of labor duration and second stage of labor duration.

**Table 3:** Relation between the use of EA, the type of delivery and the delivery duration in pregnant women who reach the second stage of labor.

Variables	Second Stage of Labor Duration				*p*-Value
	<1 h (*n* = 1577) *n* (%)	1–2 h (*n* = 547) *n* (%)	2–3 h (*n* = 357) *n* (%)	≥3 h (269)*n* (%)	
**No EA**					NC *
Normal Vaginal	476 (94.8)	18 (3.6)	6 (1.2)	2 (0.4)	
Instrumental	0 (0.0)	0 (0.0)	0 (0.0)	0 (0.0)	
C-section	0 (0.0)	0 (0.0)	0 (0.0)	0 (0.0)	
**Use of EA**					<0.001
Normal Vaginal	1049 (52.4)	495 (24.7)	281 (14.0)	177 (8.8)	
Instrumental	46 (23.7)	27 (13.9)	54 (27.8)	67 (34.5)	
CS	6 (11.5)	7 (13.5)	16 (30.8)	23 (44.2)	

CS = Caesarean Section; EA = Analgesia Epidural. * NC: Not calculable.

**Table 4:** Multivariate analysis on the use of epidural analgesia, type of delivery, duration of the second stage of labor and its link with neonatal morbidity in population 2.

Umbilical Artery pH < 7.10	Aj. OR *	CI 95%	*p*-Value
Use of EA	0.89	0.33, 2.37	0.813
Type of delivery (Ref. Normal Vaginal)			
Instrumental delivery	2.68	1.15, 6.27	0.023
C-section	2.32	0.50, 10.73	0.282
Second stage of labor duration (Ref. < 1 h)			
1–2 h	0.74	0.31, 1.78	0.497
2–3 h	0.83	0.32, 2.16	0.702
>3 h	0.81	0.48, 2.34	0.699
**Apgar at 5 min < 7**	Aj. OR *	**CI 95%**	***p*-Value**
Use of EA	NC	NC	0.933
Type of delivery (Ref. Normal Vaginal)			
Instrumental delivery	4.11	0.59, 28.70	0.154
C-section	8.23	0.68, 100.45	0.999
Second stage of labor duration (Ref. < 1 h)			
1–2 h	NC	NC	0.992
2–3 h	0.61	0.09, 4.09	0.607
>3 h	0.28	0.02, 3.28	0.311
**Advanced neonatal resuscitation**	Aj. OR *	**CI 95%**	***p*-Value**
Use of EA	NC	NC	0.993
Type of delivery (Ref. Normal Vaginal)			
Instrumental delivery	2.44	1.17, 5.08	0.017
C-section	2.56	0.71, 9.24	0.151
Second stage of labor duration (Ref. < 1 h)			
1–2 h	0.90	0.46, 1.78	0.766
2–3 h	0.66	0.29, 1.50	0.321
>3 h	0.38	0.13-1.10	0.075
**Composite morbidity**	Aj. OR *	**CI 95%**	***p*-Value**
Use of EA	1.80	0.75, 4.37	0.191
Type of delivery (Ref. Normal Vaginal)			
Instrumental delivery	2.86	1.59, 5.12	<0.001
C-section	2.20	0.73, 6.65	0.161
Second stage of labor duration (Ref. < 1 h)			
1–2 h	0.85	0.49, 1.50	0.580
2–3 h	0.73	0.38, 1.40	0.340
>3 h	0.52	0.24, 1.14	0.112

C-section = Caesarean Section; EA=Analgesia Epidural; Ref= Reference category. * Aj. OR: Odds Ratio adjusted with binary logistic regression by use of epidural analgesia, type of delivery, second stage of labor duration, maternal age, birth weight, gestational age, parity, previous caesarean section, and first stage of labor duration.
